# Satisfaction with and suitability of the problem-based learning program at the Catholic University of Korea College of Medicine

**DOI:** 10.3352/jeehp.2019.16.20

**Published:** 2019-07-19

**Authors:** Dong Mi Yoo, A Ra Cho, Sun Kim

**Affiliations:** Department of Medical Education, College of Medicine, The Catholic University of Korea, Seoul, Korea; Hallym University, Korea

**Keywords:** Problem-based learning, Medical education, Curriculum, Tutor, Republic of Korea

## Abstract

**Purpose:**

This study was conducted to identify suggestions for improving the effectiveness and promoting the success of the current problem-based learning (PBL) program at the Catholic University of Korea College of Medicine through a professor and student awareness survey.

**Methods:**

A survey was carried out by sending out mobile Naver Form survey pages via text messages 3 times in December 2018, to 44 medical students and 74 professors. In addition, relevant official documents from the school administration were reviewed. The collected data were analyzed to identify the achievement of educational goals, overall satisfaction with, and operational suitability of the PBL program.

**Results:**

The overall satisfaction scores for the PBL program were neutral (students, 3.27±0.95 vs. professors, 3.58±1.07; P=0.118). Regarding the achievement of educational goals, the integration of basic and clinical medicine and encouragement of learning motivation were ranked lowest. Many respondents expressed negative opinions about the modules (students, 25.0%; professors, 39.2%) and tutors (students, 54.5%; professors, 24.3%). The students and professors agreed that the offering timing of the program in medical school and the length of each phase were suitable, while opinions expressed in greater detail pointed to issues such as the classes being held too close to exams and their alignment with regular course units.

**Conclusion:**

Issues with modules and tutors were the most pressing. Detailed and appropriate modules should be developed on the basis of advice from professors with experience in PBL tutoring. Inconsistencies in tutoring should be reduced by standardization and retraining.

## Introduction

Medical education has moved away from traditional learning, lecture-based, and memorization-based teaching and evolved to embrace task, case, and clinical skill-based approaches to train doctors who have creative problem-solving skills using the vast array of widely-available medical knowledge in the modern world. Problem-based learning (PBL) has been reported to encourage deep learning through student-centered active learning, with learning outcomes that are achieved by promoting self-directed learning, lifelong learning capability, learning motivation, information searching and management skills, critical thinking, problem-solving, general work-related skills, collaborative learning, and interpersonal communication skills [1-3]. For this reason, PBL programs are used in many medical education settings in Korea and abroad. The Catholic University of Korea College of Medicine has also been using this method since 2009 as a way to encourage students’ learning motivation, self-directed learning, and integration of basic and clinical medicine. However, for the last decade, the focus has been on simply running the program, without a systematic review of on-site practices and educational performance. As a new curriculum is to be launched in 2021, this study was conducted to assess the program’s achievement of educational goals and the suitability of its operational implementation, based on a survey of professors’ and students’ perceptions.

## Methods

### Ethics statement

This study was approved by the Institutional Review Board (IRB) of Songeui Medical Campus, the Catholic University of Korea (IRB approval no., MC19EESI0064). The requirement to obtain informed consent was waived; however, the purpose of the survey was explained clearly and participation was completely optional. No disadvantages were given depending on participation status.

### Study design

A survey-based observational study was conducted among the medical students and professors of the Catholic University of Korea College of Medicine.

### Subjects and technical information

**Fig. 1.**
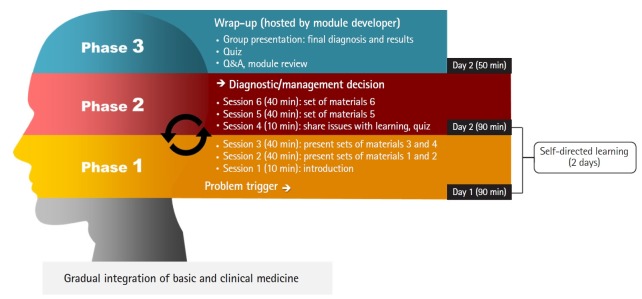
Flow chart of problem-based learning.

The PBL program is implemented in 8 unit courses for students in medical years 1 and 2 at the Catholic University of Korea College of Medicine. One module is allocated per course and tutors are required to attend an orientation by the developer of each module. The PBL program is held with 2 meetings per module, for 1.5 hours per phase, followed by a 50-minute wrap-up class instructed by the module developer, after the second phase is over (Fig. 1). With the purpose of evaluating the PBL program, a survey was conducted of PBL tutors and students who attended the programs in 2017, following the flow chart presented in Fig. 1, and relevant official documents from the school administration were reviewed (e.g., orientation materials, modules, and committee meeting minutes). The anonymous survey was distributed 3 times to target professors and students using Naver Form survey pages via text messages in December 2018 (Appendices 1, 2). Responses from 44 students and 74 tutors were used for analysis. The survey tool was developed by the research team and was completed after being reviewed by 2 medical education experts, for the purpose of collecting data needed to improve the PBL program. Responses to closed-ended questions were analyzed as mean and standard deviation (as descriptive statistics) and grouped under the criteria of achievement of educational goals, satisfaction with the PBL program, and suitability of operational implementation. Information from official documents and open-ended responses that included specific opinions were categorized according to the above criteria. The categorization was determined through the consensus of 2 researchers and 1 auditor, to prevent any bias or subjective misinterpretation. Data omission was carefully prevented as well. The raw data can be found in Supplement 1.

## Results

### Achievement of educational goals

**Fig. 2.**
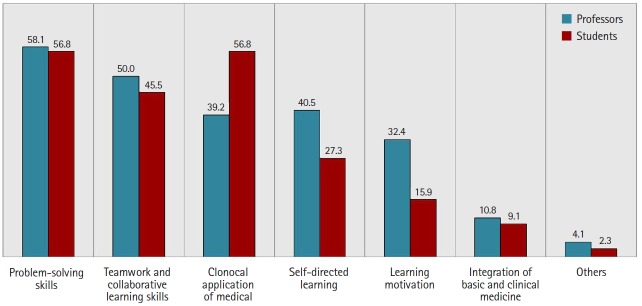
Achievement of educational goals. Values are presented as percentages.

The relevant official documents did not demonstrate any detailed and clear goals of the PBL program. The student orientation materials only presented the concepts, advantages, process, and methods of operating a PBL program in general, without proposing any desired learning outcomes. Because concrete goals were not articulated in the official documentation, this study presented respondents with well-known educational effects of PBL and asked them to choose any and all capabilities for which they had seen improvements. The results showed that they thought their capabilities had improved, and both groups selected problem solving as the most improved capability. Integration of basic and clinical medicine and encouragement of learning motivation received the lowest rank in both groups (Fig. 2).

### Satisfaction with the problem-based learning program

**Fig. 3.**
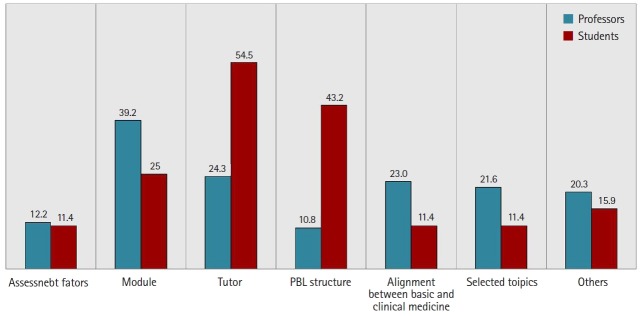
Issues in problem-based learning (PBL) class. Values are presented as percentages.

The overall satisfaction scores for the PBL program were neutral (students, 3.27±0.95 versus professors, 3.58±1.07; P=0.118). However, when asked about issues in class, the professors tended to point to inconsistencies among modules (39.2%), while the students focused on inconsistencies among tutors (54.5%) (Fig. 3). Some examples of specific problems with modules mentioned by professors are listed below.

**Professors’ opinions on modules**
“The modules were too easy and could be answered at the beginning of the session.”
“The provided materials were insufficient and caused difficulties in the session.”
“The unrefined module methods caused delays in reaching the desired performance outcomes. The modules were focused on human beings with numerous health issues, rather than focusing on a certain disease.”
“The difficulty level of the modules should be enhanced to enable students to dig deeper into the topic.”
“It may be challenging to create modules, but the level of difficulty of the modules showed a problematic degree of variation.”
“Some problems and answers had errors. They need to be revised.”
Some examples of students’ negative opinions regarding their tutors are presented below.

**Students’ opinions on tutors**
“Some tutors seem to lead the PBL session without considering that the students, who know nothing about clinical medicine. When students suggested ideas, they would reject them as ‘clinically impossible,’ which is far from the intention of PBL”
“It was difficult to grasp the learning issues since the topics were new. Tutors should intervene more when the discussions go completely off track.”
“I wish that the tutors could suggest more diverse conditions associated with certain symptoms, other than those aligned with the corresponding course unit.”
“The sessions are run in different ways depending on the tutor.”

### Suitability of operational implementation

The PBL program is offered in the fall semester of medical year 1 and throughout medical year 2. In total, 88.6% of the students agreed that the current offering timing of the program in medical school should be maintained; in contrast, 56.8% of the professors agreed that the timing should be maintained, while 27.0% wanted to expand the program. The length of the phases, with 1.5 hours per phase and 2 phases per week, was also mostly suitable (students, 72.7%; professors, 79.7%). Both groups agreed that it would be appropriate to select modules that would be more closely aligned with the content learned in the regular courses during the same period (professors: positive, 56.7% versus negative, 10.8%; students: positive, 68.2% versus negative, 9.0%). However, further discussion is necessary to make the timing of the program within the semester more appropriate. Some examples of participants’ opinions on timing are presented below.

**Professors’ opinions on timing**
“PBL program should not overlap with exam periods, when students become less interested in the classes because they must focus on their exams.”
“PBL program should be held before regular courses to promote interest.”
“The content overlap with regular course meant that students had already learned the conditions covered, leaving less room to explore new conditions.”

**Students’ opinions on timing**
“The PBL program were close to exam periods, making it difficult to prepare for the PBL.”
“Assignments were not quite relevant for the regular courses, creating a burden for other coursework and exams.”
“The PBL program was not efficient because it proceeded without basic knowledge. Arranging all PBL programs at the end of M2 would help students to have discussions more effectively and to solve problems in a more integrated way.”

## Discussion

Since the beginning of the 1990s, the major trend in medical curricula has been to integrate the teaching of basic, clinical, and psychosocial sciences [4]. Many medical schools have selected PBL as the core method to achieve these goals. PBL is known to promote motivation, enhance self-directed learning, and the integration of basic and clinical medicine, ultimately leading to long-term knowledge retention and sustained confidence [5,6].

### Problems of current problem-based learning

However, based on the feedback from students and tutors, the current PBL program of the Catholic University of Korea College of Medicine is unlikely to fulfil its original goals or purpose. First, the most serious problem was that specific goals and desired learning outcomes of the current PBL program had not been articulated in the official documentation. This lack of clarity regarding the goals of PBL appears to be the underlying factor explaining why almost no students and professors responded that integration of basic and clinical medicine had been achieved through PBL programs, and why learning motivation and self-directed learning. Second, the respondents presented a number of negative opinions on the modules and tutors. They claimed that the study materials presented in some modules were not sufficient to be useful for problem-solving and that some contained errors. Others claimed that the difficulty level was not consistent and that the performance of some tutors left room for improvement. Inappropriate tutoring failed to encourage students to solve problems, and instead involved poorly structured classes and content. Some tutors simply rejected students’ ideas or directly provided answers. Third, in terms of the operation of the program, most agreed that the offering timing of the program in medical school and length of the phases were suitable, while opinions expressed in greater detail pointed to issues such as the classes being held too close to exams and their alignment with regular course units.

### Suggestions for problem-based learning to be more effective

Based on these results, we would like to make the following suggestions. First, educational goals must be presented clearly and in detail. The goals of the PBL program must be established at the university level and conveyed to both students and tutors through various means including orientations, guidelines, and modules. Second, modules are one of the core components of the PBL program. The issues pointed out regarding the modules are serious, although the PBL Module Development Committee currently holds roughly 8 meetings annually to review and discuss the details of the PBL modules for each unit course. Amendments to the existing modules should be made in accordance with tutors’ feedback to develop well-designed modules at appropriate levels. Moreover, module development must be led by professors with experience in tutoring and the modules must be appropriate for the class structure. They should also be closely aligned with the relevant course units. Third, it is imperative to nurture and retrain PBL tutors. Tutors’ expertise and experience, social congruence, cognitive congruence, class preparedness, and tutoring technique are key factors contributing to success [7,8]. It is important to standardize tutors’ responsibilities and to provide them with suitable guidance in order to minimize inconsistencies in tutoring, and training programs should be reinforced so that tutors can provide feedback in the role of the facilitator and lead team activities that are suitable for the goals of PBL. Fourth, the current PBL program selects modules that align with regular subject matter under the official curriculum. Although this approach to module selection received positive feedback, the timing of the program within the semester worked against the success of PBL. Therefore, it would be ideal for the modules to be relevant to regular coursework and for PBL programs to be placed at earlier stages of each course unit. Specifically, the timing of the program within the semester should be reconsidered. Placing PBL programs too close to midterms and finals appears to discourage students from working hard in the PBL sessions. Students may have insufficient time for self-directed learning, which inevitably leads to unprepared classroom discussions and an excessive burden of study. Furthermore, a lack of basic knowledge on the topics covered in modules makes it difficult for students to lead in-depth discussions, and only has the effect of increasing their workload. Modules should be selected to correspond with the units covered in other courses, and classes should be arranged in consideration of other aspects of students’ academic schedule, so that basic and clinical knowledge can be more effectively integrated.

### Limitation and strength

This study was conducted based on a survey of tutors and students who taught or attended the PBL program at a single medical school with a relatively small sample size. Therefore, the generalizability of these findings is highly limited. This study is meaningful in that it reviewed a decade-old program using detailed criteria and collected feedback for future improvement.

### Conclusion

It was confirmed that PBL programs require massive resources, organization, and detailed planning, as well as consistent evaluation and management in consideration of the unique educational context of each university or college, although PBL is no longer a new method and has been adopted at most medical schools. By applying the criteria suggested in this study, it is expected that better educational results can be achieved by making efforts to improve the identified issues.

## Supplementary materials

Supplement 1. Data files are available from https://doi.org/10.7910/DVN/XZ5RVL.

Supplement 2. Audio recording of the abstract.
