# Supplementary material for: Satisfaction with and suitability of the problem-based learning program at the Catholic University of Korea College of Medicine
Source: J Educ Eval Health Prof. 2019 Jul 19;16:20. doi: 10.3352/jeehp.2019.16.20 (PMC6702123; doi:10.3352/jeehp.2019.16.20)
Supplement: Supplementary file 2 [file jeehp-16-20-app1.pdf]

Jeehp

Appendix 1. Student survey tool

Problem-based learning (PBL) experience

1. Are you satisfied in general with your experience with PBL programs?

No, not at all	1	2	3	4	5	Yes, very satisfied

2. Which of the capabilities listed below do you think you have improved through PBL? (Multiple answers are possible)

	Problem-solving skills
	Teamwork and collaborative learning skills
	Clinical application of medical knowledge
	Self-directed learning
	Learning motivation
	Integration of basic and clinical medicine
	Others

3. Which of the following do you think is a problem with the existing PBL program, if any? (Multiple answers are possible)

	Assessment factors
	Modules
	Tutors
	Structure
	Alignment between basic and clinical medicine
	Selected topics
	Others

4. The current PBL program selects module topics from content learned in regular course units. Do you think this is helpful for learning clinical knowledge and understanding actual clinical situations?

No, not at all	1	2	3	4	5	Yes, very satisfied

5. The current PBL program provides a wrap-up at the last session. Do you think the wrap-up class is helpful for understanding the relevant regular course units and the PBL module topics as a whole?

No, not at all	1	2	3	4	5	Yes, very satisfied

6. Do you think the PBL program promotes learning motivation?

No, not at all	1	2	3	4	5	Yes, very satisfied

7. Do you think the PBL program helps enhance self-directed study?

No, not at all	1	2	3	4	5	Yes, very satisfied

8. How would you rate your participation and attitude during the PBL programs?

No, not at all	1	2	3	4	5	Yes, very satisfied

PBL operational implementation

9. The current PBL program is held with 2 meetings in a week per module. Do you think this is appropriate? Please continue to 9-1 if your answer is "No".

Yes		No	

9-1. If you answered "No" to question 9, what frequency would you consider appropriate?

(   ) times per week

10. The current PBL program is offered for 1.5 hours per phase. Do you think this is appropriate? Please continue to 10-1 if your answer is "No"

Yes		No	

10-1. If you answered "No" to question 10, what length would you consider appropriate?

(   ) minutes per phase

11. The current PBL program is offered in the fall semester of medical year 1 and throughout medical year 2. What do you think would be the most appropriate time to offer the PBL program for it to be effective? Please answer 11-1 if you do not think the current timing of the program in medical school is appropriate.

Maintain the current status:<br>3 semesters; medical year 1 fall semester to medical year 2 fall semester	
Prefer different timing (continue to 11-1)	

11-1. Which years do you think would be appropriate for a PBL program? (Multiple answers are possible)

	Premedical year 1
	Premedical year 2
	Medical year 1
	Medical year 2
	Medical year 3
	Medical year 4

12. Please specify any positive opinions that you have about the PBL program.

13. Please specify any improvements that you would suggest for the PBL program.

