# Supplementary material for: Satisfaction with and suitability of the problem-based learning program at the Catholic University of Korea College of Medicine
Source: J Educ Eval Health Prof. 2019 Jul 19;16:20. doi: 10.3352/jeehp.2019.16.20 (PMC6702123; doi:10.3352/jeehp.2019.16.20)
Supplement: Supplementary file 3 [file jeehp-16-20-app2.pdf]

Appendix 2. Professor survey tool

Problem-based learning (PBL) experience

1. Are you satisfied in general with your experience with PBL programs?

No, not at all	1	2	3	4	5	Yes, very satisfied

2. Which of the student capabilities listed below do you think you have improved through PBL? (Multiple answers are possible)

	Problem-solving skills
	Teamwork and collaborative learning skills
	Clinical application of medical knowledge
	Self-directed learning
	Learning motivation
	Integration of basic and clinical medicine
	Others

3. Which of the following do you think is a problem with the existing PBL program, if any? (Multiple answers are possible)

	Assessment factors
	Modules
	Tutors
	Structure
	Alignment between basic and clinical medicine
	Selected topics
	Others

4. The current PBL program selects module topics from content learned in regular course units. Do you think this is helpful for learning clinical knowledge and understanding actual clinical situations?

No, not at all	1	2	3	4	5	Yes, very satisfied

5. Do you think the PBL program promotes students' learning motivation?

No, not at all	1	2	3	4	5	Yes, very satisfied

6. Do you think the PBL program promotes students' self-directed study ability?

No, not at all	1	2	3	4	5	Yes, very satisfied

7. How were the overall participation and attitudes of students in the PBL program?

No, not at all	1	2	3	4	5	Yes, very satisfied

PBL operational implementation

8. The current PBL program is held with 2 meetings in a week per module. Do you think this is appropriate? Please continue to 8-1 if your answer is "No".

Yes		No	

8-1. If you answered "No" to question 8, what frequency would you consider appropriate?

( ) times per week

9. The current PBL program is offered for 1.5 hours per phase. Do you think this is appropriate? Please continue to 9-1 if your answer is "No".

Yes		No	

9-1. If you answered "No" to question 10, what length would you consider appropriate?

( ) minutes per phase

10. The current PBL program is offered in the fall semester of medical year 1 and throughout medical year 2. What do you think would be the most appropriate time to offer the PBL program for it to be effective?

Maintain the current status:<br>3 semesters; medical year 1 fall semester to medical year 2 fall semester	
Prefer different timing (continue to 11-1)	
Reduce the PBL program (continue to 10-2)	

10-1. Which years do you think would be appropriate for a PBL program? (Multiple answers are possible)

	Premedical year 1
	Premedical year 2
	Medical year 1
	Medical year 2
	Medical year 3
	Medical year 4

10-2. Reduction: From which years do you think the PBL program should be removed? (Multiple answers are possible)

	Premedical year 1
	Premedical year 2
	Medical year 1
	Medical year 2
	Medical year 3
	Medical year 4

11. What do you think should be done as a priority for the PBL program to achieve its goals and operate more effectively? (Multiple answers are possible. Select up to 2 answers)

	Improve tutor training
	Policy support, such as mandating tutor training and introducing performance assessments
	Stronger alignment between module topics and course units
	Independent arrangement of a PBL program regardless of course units
	Changes in lecture structure and methods
	Improvement of student assessment methods
	Others

12. Please specify any positive opinions that you have about the PBL program.

13. Please specify any improvements that you would suggest for the PBL program.

